# Knowledge, willingness, uptake and barriers of cervical cancer screening services among Chinese adult females: a national cross-sectional survey based on a large e-commerce platform

**DOI:** 10.1186/s12905-023-02554-2

**Published:** 2023-08-17

**Authors:** Bo Zhang, Sumeng Wang, Xiyu Yang, Mingyang Chen, Wenhui Ren, Yanping Bao, Youlin Qiao

**Affiliations:** 1 School of Population Medicine and Public Health, Chinese Academy of Medical Sciences & Peking Union Medical College, Beijing, China; 2 National Cancer Center/National Clinical Research Center for Cancer/Cancer Hospital, Chinese Academy of Medical Sciences & Peking Union Medical College, Beijing, China; 3 The High School Affiliated to Renmin University of China, Beijing, China; 4 National Institute On Drug Dependence and Beijing Key Laboratory of Drug Dependence, Peking University, Beijing, China; 5 Present Address: School of Population Medicine and Public Health, Chinese Academy of Medical Sciences and Peking Union Medical College, Beijing, 100005 China

**Keywords:** Cervical cancer, Cancer screening, Knowledge, Awareness, Practice

## Abstract

**Background:**

Improving the coverage rate of cervical cancer screening is a challenge mission for cervical cancer elimination. This study attempted to assess the knowledge, willingness, and uptake of cervical cancer screening services among Chinese females and determined associated factors.

**Methods:**

This is a cross-sectional online survey conducted in China from March to April 2022. Information on demographic characteristics, knowledge, willingness, and uptake of cervical cancer screening was collected through a large e-commerce platform. Women aged 18–65 were included in the analysis. Logistic regression analysis was employed to detect the possible factors associated with knowledge, willingness, and screening participation.

**Results:**

A total of 4518 women (37.83 ± 9.14 years) were included in the final analysis, of whom 87.16% (*n* = 3938) lived in urban areas. About 93.40% (*n* = 4220) of the respondents reported hearing of cervical cancer screening. The median score of knowledge about cervical cancer was 16 out of 26. Over 84% (*n* = 3799) of the respondents were willing to receive regular cervical cancer screening. Nearly 40% (*n* = 1785) had never received cervical cancer screening. Among the screened women, 21.26% (*n* = 581), 35.24% (*n* = 1151), and 42.37% (*n* = 1158) were screened through a national cervical cancer screening program, employee physical examination, and self-paid physical examination, respectively. Knowledge was positively associated with willingness and screening participation. Age, marital status, occupation, monthly household income, and HPV vaccination history could influence screening participation (all *p* < 0.05).

**Conclusions:**

Though women had high-level awareness and strong participation willingness in cervical cancer screening, the overall screening coverage among Chinese women was still low. Besides, the knowledge about cervical cancer was still limited. Comprehensive health education should be enhanced by utilizing social media platforms and medical workers. It is also important to promote national free cervical cancer screening with high-performance screening methods.

## Introduction

Cervical cancer has been acknowledged as the first malignant tumor that can be effectively prevented. Apart from the human papillomavirus (HPV) vaccine, cervical cancer screening is considered highly cost-effective for preventing cervical cancer, through the early diagnosis and management of the precursor lesions [1–5]. Since the non-HPV-associated cervical cancer, insufficient supply of HPV vaccine, and the incidence risk among women previously contracting oncogenic HPV types, improving access to screening is a major priority of the global elimination of cervical cancer [4, 6]. In 2020, the World Health Organization (WHO) proposed a strategy to accelerate the global elimination of cervical cancer, with an objective of having 70% of women screened using a high-performance test by the ages of 35 and 45 years by 2030 [6]. One study shows that countries with population-based and high-quality screening have made great progress in decreasing incidence rates and mortality rates for cervical cancer (e.g., the UK, Sweden, and Australia) [7]. However, the provision of screening services in most low- and middle-income countries (LMICs) are still insufficient. About 35% of low-income and less than 55% of lower-middle-income countries had a national cervical cancer screening program (NCCSP) in 2020, far below the proportion of more than 80% in high-income countries [6]. Data shows that about 90% of the new cases and deaths of cervical cancer in 2020 exist in LMICs [8]. Therefore, the adoption and expansion of screening in LMICs are crucial for the global elimination of cervical cancer.

As one of the LMICs with a sizable population, China accounts for almost 1/5 of the worldwide cervical cancer burden [9]. In China, age-standardized incidence and mortality of cervical cancer attributed to HPV were 10.42 and 2.84 per 1,00,000 respectively [10]. Chinese women receive screening through organized screening (e.g., free screening programs organized by the government and employee physical examinations paid by employers) and opportunistic screening (e.g., self-paid examinations in outpatient service). Compared with opportunistic screening, organized screening is considered to be superior in diminishing socioeconomic inequalities, increasing screening rates, and enhancing follow-up [11–13]. In 2009, China initiated the National Cervical Cancer Screening Program (NCCSP), offering free screening for 10 million women with rural household registration between the age of 35 and 64 each year [14, 15]. Yet, a large number of urban women lacked access to free cervical cancer screening until 2022 when NCCSP was available to women with urban household registration. Given that China is still promoting urbanization and a large number of rural people migrate to cities, it is an urgent need to improve the coverage of cervical cancer screening among urban females.

The participation of cervical cancer screening is largely decided by people’s knowledge and willingness. About 34% of the Chinese women aged between 30 and 49 years have received cervical cancer screening, with 130 million women at this age stage having never been screened [16]. This may be related to the low knowledge, awareness and acceptance of cervical cancer screening among women [17]. To our knowledge, all published studies on women’s knowledge, awareness and practice towards cervical cancer screening in China were regionally based.

In order to evaluate the present status of knowledge, awareness, and participation of cervical cancer screening in China, we performed this nationwide survey. Findings from this study could provide scientific basis for effective promotion of women’s cervical cancer screening policy and women’s health education.

## Methods and materials

### Study design and population

We conducted a nationwide cross-sectional network survey in mainland China from March 5 to April 7 of 2022. Individuals who understand the survey and were voluntarily participate were eligible to recruitment. Only women aged 18–65 years were included into the analysis. The online questionnaire was distributed via the Chinese website Joybuy (JD.com, Inc., Beijing, China), a online commerce platform with health information service. This survey was based on convenience sampling. The registered members of Joybuy who browsed the website and clicked the link of survy were instructed to complete the online questionnaire.

The study was approved by the research ethics committee of the Chinese Academy of Medical Sciences and Peking Union Medical College (approval number CAMS&PUMC-IEC-2022–020). The survey was conducted anonymously and the informed consents were obtained at the start of the survey.

### Questionnaire

The questionnaire was designed under the guidance of 2 experts in cervical cancer prevention, referring to the previous study. We conducted a pilot survey on 40 women. Then the questionnaire was modified referring to the feedback of pilot survey. The questionnaire covered 4 domains: (i) Socio-demographic characteristics, such as age, ethnicity, places of residence, marital status, etc. (ii) Awareness and knowledge on cervical cancer, HPV vaccine and cervical cancer screening. Questions in this section referred to the Work Plan for Cervical Cancer Screening of the National Health Commission. (iii) Willingness to cervical cancer screening and obstacles to get screened. (iv) The behavior of cervical cancer screening, including time of last screening, methods of previous screening, etc.

### Statistical analysis

Descriptive statistics were performed for socio-demographic characteristics, awareness, and screening behaviors by number and percentages. Knowledge about cervical cancer was assessed using knowledge score. One point was assigned to each correct option. For questions with multiple correct options, no points was given once a wrong choice was selected. The respondents were classified into low- and high-score group according to the overall knowledge median score. Univariate and multivariate logistic regressions were used to identify factors associated with knowledge, willingness and uptake of cervical cancer screening. All statistical analyses were performed using R software (version 4.1.0; R Foundation for Statistical Computing, Vienna, Austria), with a 0.05 *p* value used to determine statistical significance.

## Results

### Demographics

Of the 8753 individuals who browsed the questionnaire, 5459 signed an informed consent and completed the questionnaire, with a response rate of 62.53%. In addition, 941 respondents were excluded due to male (*n* = 845) or beyond the target age range (*n* = 96). Table 1 shows the demographic characteristics of the respondents included. A total of 4518 women whose mean age were 37.83 ± 9.14 years were included into the analysis. Around 87.16% lived in urban areas and 81.32% were married. Over 75% holding college degrees or above. Only 7.5% of the respondents were medical workers and 36.50% were employees in companies. Nearly 62% had a monthly household income of “6001–20000 RMB”. About 23.28% of the respondents had received HPV vaccination.

**Table 1:** Characteristics of the respondents

Characteristics	N	%
Age group		
18–30	982	21.73
31–35	1073	23.75
36–40	831	18.39
41–45	687	15.21
46–50	467	10.34
51–65	478	10.58
Residence		
Rural	580	12.84
Urban	3938	87.16
Marital status		
Unmarried^a^	844	18.68
Married	3674	81.32
Education		
High school and below	1111	24.59
College and above	3407	75.41
Occupation		
Medical worker	344	7.61
Student	140	3.10
Worker or farmer	222	4.91
Government staff	554	12.26
Employee in company	1649	36.50
Service industry staff	651	14.41
Unemployed	191	4.23
Other	767	16.98
Monthly household income (RMB)^b^		
< 6000	1000	22.13
6001 ~ 10,000	1489	32.96
10,001 ~ 20,000	1310	29.00
> 20,000	719	15.91
History of chronic diseases		
No	3821	84.57
Yes	697	15.43
HPV vaccination history		
No	3466	76.72
Yes	1052	23.28
**Total**	4518	100.00

^a^The unmarried category included separated, divorced, and widowed

^b^As of 30 Nov 2022, 1 RMB = USD $0.14

### Awareness and knowledge

**Fig. 1:**
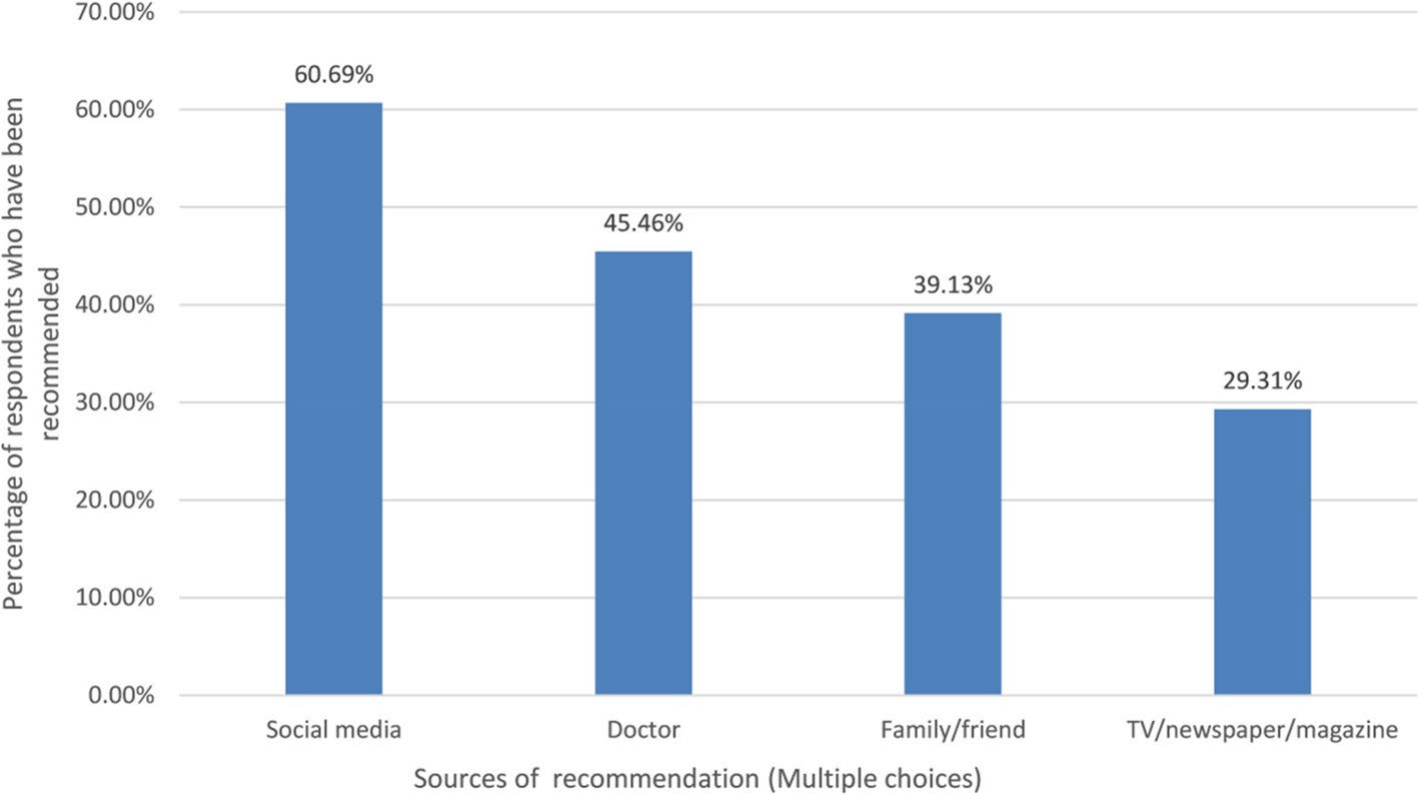
Sources of recommendation (Multiple choices)

In our study, a large majority had heard of cervical cancer screening (93.40%, *n* = 4220) and HPV vaccine (92.87%, *n* = 4196). About 59.92% (*n* = 2707) had heard of the global strategy to accelerate the elimination of cervical cancer proposed by WHO. As illustrated in Fig. 1, the main sources of information about cervical cancer screening were social media (60.69%, *n* = 2742) and advice from doctors (45.46%, *n* = 2054). The median score of knowledge was 16 out of 26. Of those who had heard of cervical cancer screening, about 75.87% (3202/4220) knew that women aged 35–64 years should receive regular cervical cancer screening, and 72.25% (3049/4220) knew the screening interval.

Table 2 shows the factors relative with the knowledge about cervical cancer. In multivariate logistic regression analysis, women with a college degree or above tended to have a higher level of knowledge (aOR = 1.684, 95% CI: 1.430–1.984). Medical workers were reported higher knowledge levels than those with any other occupation. The women with more monthly family income tended to have higher levels of knowledge (compared with < 6000 RMB, aOR = 1.453 [95% CI: 1.224–1.726] for 6001–10000 RMB; aOR = 1.818 [95% CI: 1.513–2.184] for 10001 ~ 20000 RMB; aOR = 1.948 [95%CI: 1.570–2.416] for > 20000 RMB). Other factors associated with knowledge on cervical cancer were 51–65 years old (aOR = 0.766, 95% CI: 0.597–0.981), having chronic diseases (aOR = 0.805, 95% CI: 0.677–0.957), and HPV vaccination (aOR = 1.566, 95% CI: 1.347–1.821).

**Table 2:** Associated factors of knowledge level about cervical cancer

**Characteristics**	**High knowledge level N (%)**	**Odds ratio**			
		**Crude (95% CI)**	***p* value**	**Adjusted (95% CI)**	***p* value**
Age group					
18–30	565 (22.87)	1		1	
31–35	608 (24.61)	0.965 (0.810–1.149)	0.690	0.985 (0.816–1.190)	0.878
36–40	473 (19.14)	0.975 (0.809–1.175)	0.792	1.051 (0.859–1.286)	0.627
41–45	368 (14.89)	0.851 (0.700–1.036)	0.108	0.920 (0.744–1.139)	0.445
46–50	248 (10.04)	0.836 (0.670–1.043)	0.112	1.056 (0.830–1.344)	0.656
51–65	209 (8.46)	0.573 (0.460–0.715)	< 0.001	0.766 (0.597–0.981)	0.035
Residence					
Rural	268 (10.85)	1		1	
Urban	2203 (89.15)	1.478 (1.241–1.761)	< 0.001	1.088 (0.895–1.322)	0.397
Marital status					
Unmarried	485 (19.63)	1			
Married	1986 (80.37)	0.871 (0.749–1.013)	0.073		
Education					
High school and below	430 (17.40)	1		1	
College and above	2041 (82.60)	2.366 (2.060–2.719)	< 0.001	1.684 (1.430–1.984)	< 0.001
Occupation					
Medical worker	261 (10.56)	1		1	
Student	90 (3.64)	0.572 (0.374–0.875)	0.010	0.580 (0.370–0.908)	0.017
Worker or farmer	83 (3.36)	0.190 (0.132–0.274)	< 0.001	0.375 (0.253–0.556)	< 0.001
Government staff	320 (12.95)	0.435 (0.322–0.586)	< 0.001	0.423 (0.312–0.573)	< 0.001
Employee in company	933 (37.76)	0.414 (0.318–0.540)	< 0.001	0.415 (0.317–0.544)	< 0.001
Service industry staff	342 (13.84)	0.352 (0.263–0.471)	< 0.001	0.459 (0.339–0.621)	< 0.001
Unemployed	76 (3.08)	0.210 (0.144–0.308)	< 0.001	0.348 (0.234–0.518)	< 0.001
Other	366 (14.81)	0.290 (0.218–0.386)	< 0.001	0.409 (0.305–0.549)	< 0.001
Monthly household income					
< 6000	402 (16.27)	1		1	
6001 ~ 10,000	807 (32.66)	1.760 (1.496–2.071)	< 0.001	1.453 (1.224–1.726)	< 0.001
10,001 ~ 20,000	799 (32.34)	2.326 (1.966–2.752)	< 0.001	1.818 (1.513–2.184)	< 0.001
> 20,000	463 (18.74)	2.690 (2.207–3.280)	< 0.001	1.948 (1.570–2.416)	< 0.001
Chronic diseases					
No	2136 (86.44)	1		1	
Yes	335 (13.56)	0.730 (0.621–0.858)	< 0.001	0.805 (0.677–0.957)	0.014
HPV-vaccinated					
No	1778 (71.95)	1		1	
Yes	693 (28.05)	1.833 (1.587–2.116)	< 0.001	1.566 (1.347–1.821)	< 0.001

*CI* Confidence interval

### Willingness

**Fig. 2:**
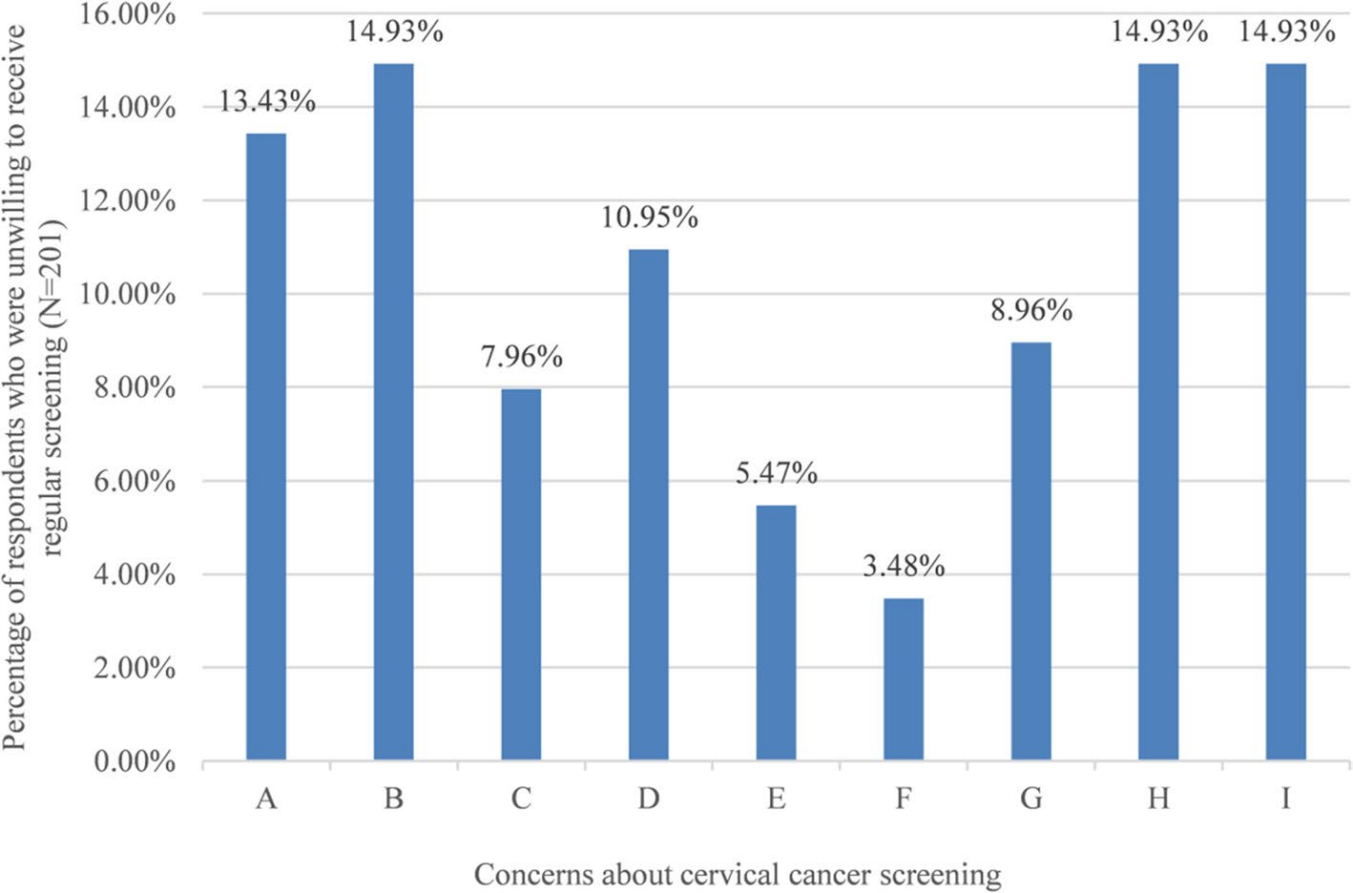
Respondents’ concerns about cervical cancer screening. A: I have no sexual life so I have no risk of HPV infection; B: I am in fear of pain; C: I worried about the abnormal results; D: I have no cervical symptoms of at present; E: I am afraid of being cheated; F: No time or inconvenient to have a screening; G: I feel embarrassed or even humiliated to be screened.; H: I have no idea about the benefits of inspection; I: I do not want to go to hospitals

Over 84% (3799/4518) of the respondents were willing to receive regular cervical cancer screening. About 12.86% (*n* = 581) indicated that their willingness would depend on the results of the previous examination. As shown in Fig. 2, among the women who were unwilling to receive regular screening (0.03%, *n* = 138), the obstacles for women to participate in regular screening included “Fear of pain” (14.93%, *n* = 30), “Don’t want to visit hospital” (14.93%, *n* = 30), “No idea about the benefit of screening” (14.93%, *n* = 30), “without risk of HPV infection because no sexual” (13.43%, *n* = 27), etc.

Table 3 shows the factors associated with the willingness to participate in regular cervical cancer screening. Multivariate logistic regression analysis showed that willingness was associated with marital status, occupation, monthly household income, previous HPV vaccination, and score of knowledge about cervical cancer. The higher score of knowledge indicated more willingness to participate in screening (aOR = 1.132, 95% CI: 1.114–1.151). Women who were married (aOR = 1.821, 95% CI: 1.444–2.296), and who had a monthly family income of “6001–10000 RMB” (aOR = 1.278, 95% CI: 1.025–1.595) or “ > 20000 RMB” (aOR = 2.044, 95% CI: 1.476–2.831) were more willing to participate in regular cervical cancer screening. Furthermore, women who were unemployed were less willing compared to medical workers.

**Table 3:** Associated factors of willingness to regular cervical cancer screening

**Characteristics**	**Willing to regular screening N (%))**	**Odds ratio**			
		**Crude (95% CI)**	***p* value**	**Adjusted (95% CI)**	***p* value**
Age group					
18–30	801 (21.08)	1		1	
31–35	925 (24.35)	1.412 (1.114–1.790)	0.004	1.117 (0.853–1.464)	0.422
36–40	720 (18.95)	1.466 (1.134–1.895)	0.004	1.206 (0.898–1.618)	0.213
41–45	577 (15.19)	1.185 (0.914–1.537)	0.200	1.002 (0.740–1.357)	0.989
46–50	406 (10.69)	1.504 (1.099–2.058)	0.011	1.403 (0.981–2.008)	0.064
51–65	370 (9.74)	0.774 (0.592–1.012)	0.061	0.825 (0.594–1.146)	0.251
Residence					
Rural	463 (12.19)	1		1	
Urban	3336 (87.81)	1.400 (1.123–1.746)	0.003	0.949 (0.736–1.225)	0.690
Marital status					
Unmarried	659 (17.35)	1		1	
Married	3140 (82.65)	1.651 (1.369–1.990)	< 0.001	1.821 (1.444–2.296)	< 0.001
Education					
High school and below	867 (22.82)	1		1	
College and above	2932 (77.18)	1.737 (1.463–2.063)	< 0.001	1.021 (0.822–1.268)	0.852
Occupation					
Medical worker	315 (8.29)	1		1	
Student	111 (2.92)	0.352 (0.202–0.616)	< 0.001	0.642 (0.350–1.177)	0.152
Worker or farmer	174 (4.58)	0.334 (0.203–0.548)	< 0.001	0.705 (0.407–1.221)	0.212
Government staff	490 (12.90)	0.705 (0.445–1.118)	0.137	0.879 (0.545–1417)	0.596
Employee in company	1408 (37.06)	0.538 (0.359–0.806)	0.003	0.683 (0.449–1.038)	0.074
Service industry staff	543 (14.29)	0.463 (0.300–0.714)	< 0.001	0.710 (0.449–1,121)	0.141
Unemployed	141 (3.71)	0.260 (0.158–0.427)	< 0.001	0.479 (0.281–0.818)	0.007
Other	617 (16.24)	0.379 (0.249–0.576)	< 0.001	0.633 (0.406–0.986)	0.043
Monthly household income					
< 6000	756 (19.90)	1		1	
6001 ~ 10,000	1258 (33.11)	1.758 (1.437–2.150)	< 0.001	1.278 (1.025–1.595)	0.029
10,001 ~ 20,000	1128 (29.69)	2.000 (1.617–2.475)	< 0.001	1.253 (0.982–1.598)	0.070
> 20,000	657 (17.29)	3.420 (2.539–4.606)	< 0.001	2.044 (1.476–2.831)	< 0.001
Chronic diseases					
No	3235 (85.15)	1		1	
Yes	564(14.85)	0.768 (0.624–0.946)	0.013	0.848 (0.675–1.066)	0.157
HPV-vaccinated					
No	2867 (75.47)	1		1	
Yes	932 (24.53)	1.623 (1.316–2.001)	< 0.001	1.218 (0.972–1.527)	0.086
Knowledge score		1.144 (1.127–1.162)	< 0.001	1.132-(1.114–1.151)	< 0.001

*CI* Confidence interval

### Screening participation

Nearly 40% of the respondents (1785/4518) had never received any cervical cancer screening. Around 3.74% (169/4518) had postponed or cancelled cervical cancer screening due to COVID-19. Among women who received screening previously (60.49%, *n* = 2733), around 82.14% (*n* = 2245) had been screened within 3 years. About 42.37% (1158/2733) of the respondents had been screened by out-of-pocket money, 35.24% (963/2733) received screening paid by the employers, and only 21.26% (581/2733) participated in the NCCSP organized by government. Regarding the methods of examination, 61% (1667/2733) reported they had undergone cervical cytology, which was followed by high-risk HPV DNA test (42.11%, 1151/2733), colposcopy (35.35%, 966/2733), and VIA/VILI (visual inspection with acetic acid/visual inspection with Lugol’s iodine) (14.64%, 400/2733). About 2.56% of the screened women (70/2733) had abnormal screening results with only 24.29% (17/70) received treatments.

Table 4 shows the factors relative with cervical cancer screening participation. In multivariate logistic regression analysis, age was positively associated with the participation of cervical cancer screening. The married women were almost 3 times more likely to participate screening (aOR = 3.079, 95% CI: 2.542–3.729). Students (aOR = 0.281, 95% CI: 0.167–0.475), workers or farmers (aOR = 0.623, 95% CI: 0.415–0.935), and the unemployed (aOR = 0.503, 95% CI: 0.335–0.756) were less likely to participate in a screening when compared with medical workers. The women who had received HPV vaccination were 2.7 times more likely to participate in cervical cancer screening (aOR = 2.709, 95% CI: 2.273–3.228). Other factors relative with cervical cancer screening participation were monthly household income of “10001–20000 RMB” (aOR = 1.306, 95% CI: 1.071–1.591) or “ > 20000 RMB” (aOR = 1.272, 95% CI: 1.008–1.605), and score of knowledge (aOR = 1.090, 95% CI: 1.076–1.104).

**Table 4:** Associated factors of cervical cancer screening participation

**Characteristics**	**Received screening before N (%)**	**Odds ratio**			
		**Crude (95% CI)**	***p* value**	**Adjusted (95% CI)**	***p* value**
Age group					
18–30	432 (15.81)	1		1	1
31–35	629 (23.02)	1.804 (1.514–2.149)	< 0.001	1.188 (0.968–1.459)	0.100
36–40	533 (19.50)	2.277 (1.884–2.753)	< 0.001	1.527 (1.223–1.907)	< 0.001
41–45	470 (17.20)	2.758 (2.248–3.383)	< 0.001	1.931 (1.520–2.454)	< 0.001
46–50	336 (12.29)	3.265 (2.574–4.143)	< 0.001	2.488 (1.892–3.270)	< 0.001
51–65	333 (12.18)	2.924 (2.318–3.688)	< 0.001	2.536 (1.923–3.345)	< 0.001
Residence					
Rural	285 (10.43)	1		1	1
Urban	2448 (89.57)	1.701 (1.427–2.026)	< 0.001	1.199 (0.975–1.475)	0.085
Marital status					
Unmarried	302 (11.05)	1		1	1
Married	2431 (88.95)	3.510 (3.002–4.104)	< 0.001	3.079 (2.542–3.729)	< 0.001
Education					
High school and below	639 (23.38)	1		1	1
College and above	2094 (76.62)	1.178 (1.027–1.352)	0.02	0.851 (0.712–1.018)	0.077
Occupation					
Medical worker	245 (8.96)	1		1	1
Student	28 (1.02)	0.101 (0.063–0.163)	< 0.001	0.281 (0.167–0.475)	< 0.001
Worker or farmer	122 (4.46)	0.493 (0.346–0.701)	< 0.001	0.623 (0.415–0.935)	0.022
Government staff	376 (13.76)	0.854 (0.636–1.145)	0.291	0.908 (0.660–1.249)	0.552
Employee in company	1043 (38.16)	0.695 (0.539–0.897)	< 0.001	0.834 (0.633–1.100)	0.200
Service industry staff	382 (13.98)	0.574 (0.433–0.760)	< 0.001	0.735 (0.538–1.004)	0.053
Unemployed	84 (3.07)	0.317 (0.219–0.459)	< 0.001	0.503 (0.335–0.756)	0.001
Other	453 (16.58)	0.583 (0.443–0.767)	< 0.001	0.696 (0.513–0.944)	0.020
Monthly household income					
< 6000	499 (18.26)	1		1	1
6001 ~ 10,000	894 (32.17)	1.509 (1.283–1.773)	< 0.001	1.179 (0.981–1.416)	0.079
10,001 ~ 20,000	860 (31.47)	1.919 (1.621–2.271)	< 0.001	1.306 (1.071–1.591)	0.008
> 20,000	480 (17.56)	2.016 (1.653–2.459)	< 0.001	1.272 (1.008–1.605)	0.043
Chronic diseases					
No	2273 (83.17)			1	1
Yes	460 (16.83)	1.322 (1.116–1.566)	0.001	1.195 (0.988–1.444)	0.066
HPV-vaccinated					
No	1954 (71.50)	2.208 (1.894–2.574)	< 0.001	1	1
Yes	779 (28.50)			2.709 (2.273–3.228)	< 0.001
Knowledge score		1.086 (1.074–1.099)	< 0.001	1.090 (1.076–1.104)	< 0.001

*CI* Confidence interval

## Discussion

This is a nationwide survey with broad geographic coverage in mainland China, through one of the largest e-commerce platforms in China. We assessed the level of knowledge, willingness and practices related to cervical cancer screening among Chinese females and identified influencing variables. The awareness and willingness of cervical cancer screening were up to 93% and 84%, respectively. However, only 60% of the women have received cervical cancer screening, which was below the target of the WHO strategy. Knowledge was positively associated with both women’s willingness and participation in cervical cancer screening. Other factors related to screening participation included age, marital status, industry of employment, occupation, household income, and HPV vaccination.

In this study, most respondents lived in urban area. This may be because our online questionnaire is distributed via a platform that primarily consists of urban users The NCCSP in China was only available to women with rural household registration until 2022, leaving out a large number of women with urban household registration. In order to benefit more women, the NCCSP plan to cover urban women aged 35–64 years from 2022, giving priority to urban women living on subsistence allowances. Therefore, it is necessary to pay more attention to Chinese urban women about their awareness, knowledge, willingness and current status on cervical cancer screening.

About 60% of the respondents in our study has received cervical cancer screening at least once, which is nearly equal to that in Shenzhen [18] and rural areas with NCCSP [19]. Even if NCCSP was unavailable in the cities before, the screening coverage rate of urban women is no worse than that of rural areas. Only about 21% of the women have ever participated in NCCSP, which may be because most women in this study lived in cities. Worryingly, our results showed that only a minority group has participated in the NCCSP even in rural women. Except NCCSP, urban women were screened mainly through employee physical examination and self-paid physical examination. However, a large number of unemployed women and housewives were ineligible to employee physical examination, and may be also not willing to participate in screening at their own expense. These vulnerable individuals who lack access to screening should be the focus of NCCSP in the future. From 2022, urban women are eligible to NCCSP, and the cervical cancer screening rate of urban women were expected to be improved.

We found that knowledge about cervical cancer was positively associated with both willingness and participant of cervical cancer screening. This finding was also observed in some cross-sectional studies. A study conducted among 8639 women demonstrated that high HPV-related knowledge level was significantly associated with HPV testing behavior [20]. Likewise, Liu et al. reported that related knowledge was higher among the screened group compared with the unscreened group [19]. Another study by Setiawan et al. discovered that the willingness to screening was significantly affected by HPV knowledge [21]. It is widely believed that the knowledge about cervical cancer among general population might influence the practice of seeking relevant health care. However, we also found that the level of knowledge about HPV and cervical cancer was still limited even on some basic aspects. For example, approximately 30% of the respondents had no idea about the screening age and the screening interval. And nearly 16% of the respondents believed there is no risk of cervical cancer after HPV vaccination. The lack of knowledge may adversely affects the promotion of cervical cancer screening, which suggests an urgent need to strengthen public health education about the knowledge of cervical cancer prevention.

In our study, the women who were unwilling to receive regular screening reflected their unscientific notion that they are in no need of screening since “normal results of previous examination”, “no sexual life”, “no symptoms related to cervical cancer” and “menopause”. Some women believed that no symptoms or pain meant healthy, or that postmenopausal women would not develop cervical cancer. This finding indicated that merely publicizing the benefits of cervical cancer screening is not enough. Tailored health education about HPV and cervical cancer should be carried out for targeted populations, so as to help women increase their comprehensive awareness of cancer and to improve their ability to recognize their personal health.

Besides, awareness of cervical cancer screening was related to the uptake of screening [17]. A systematic review reported that the lack of awareness about the screening was the most common barrier to cervical cancer screening in LMICs [22]. Encouragingly, we found that a large majority of the women were aware of HPV and cervical cancer screening, which is consistent with the study in Shenzhen [18, 23]. We also found that the awareness about cervical cancer screening in urban China was improved when compared with a similar survey in 2008 [24]. The increased awareness may be attributed to the efforts to strengthen health education and improve cervical cancer screening coverage by the government and medical institutions. Notably, the awareness of cervical cancer varies greatly between women in rural and urban areas. Zhao et al. found that the awareness among rural women was significantly lower than urban women (46.87% vs.84.99%) [23], indicating that the awareness of Chinese women may be lower than the results of our study.

Social-economic factors may also influence the uptake of medical services. Women with household incomes were more likely to receive screening service. This may be because they are more informed about medical issues or they have more access to healthcare services. The screening rate among women who had received HPV vaccination was higher than that among those unvaccinated. This is consistent with a survey conducted in 2260 female and may be because vaccinated women are more health-conscious [25]. In addition, several studies suggested that other factors may influence the action to screening, including social support, distrust of medical institutions, culture differences, cost of screening, geographical traffic considerations [25–28]. These could explain the discrepancy between high willingness and the actual screening practice.

It is essential to utilize high-performance techniques when expanding cervical cancer screening. Although VIA/VILI is simple and low-cost, it has been phased out due to the high false negative rate observed in China [29]. For cytology, pap smear method is transitioning to TCT (thinprep cytologic test) due to the high risk of false positive and false negative results. In terms of HPV test, high-risk HPV test reportedly have higher sensitivity than cytology [30–32]. NCCSP should incorporate high-risk HPV tests according to the findings by Zhang et al. [31]. Currently, cytology is used in NCCSP in majority of Chinese regions, while HPV test is conducted only in pilot areas as the joint screening methods (HPV test + cytology). The cervical cancer screening methods and strategies are not unified in China because of the different economic and medical levels in different regions. When making screening strategies, cost and effectiveness must be carefully considered and ensure the quality of early detection.

Social media/internet and doctors were the main sources of the information on HPV or cervical cancer screening [33–35]. On the one hand, this study indicated that public awareness and knowledge of health issues may be effectively increased through social media. To increase screening coverage, the government and medical institutions might advertise cervical cancer screening through the network. On the other hand, issues regarding the internet’s role in the spread of false information and unwarranted fear should be brought up. It is also important to highlight that there are still a significant number of people who obtain their information mostly through suggestions made by friends, family, or local physicians. There are lots of women who are illiterate or can not understand mandarin Chinese, especially in rural areas. That means it was difficult for these women to receive health information about cervical cancer screening from media/internet. Therefore, it is still important to encourage clinicians to advise women to get screened for cervical cancer during consultations.

Lack of access to cervical cancer screening may be another barrier in rural areas. China has proposed the “Action Plan for Accelerating the Elimination of Cervical Cancer (2023–2030)”. In order to increase the screening coverage rate among eligible women, especially for those who have never undergone the screening, the action plan mentions some feasible measures, including promotion of the appointment system for cervical cancer screening, reasonable layout of screening sites and mobile screening units, thereby increasing access to cervical cancer screening. Further research on cost-effectiveness of the implementation of these measures in rural areas is needed.

Additionally, our research reveals that only a small percentage of women with abnormal screening results ever received treatments. Presently, radical hysterectomy, pelvic node dissection, radiotherapy, chemotherapy, or a combination of such treatments are considered to be the primary treatment options [36–38]. In general, early detection and treatment of cervical cancer result in better outcomes. Patients with early-stage cervical cancers have high survival rates. Furthermore, attention should be paid to recurrence after treatments. For example, recurrent cervical cancer after surgery may be related to factors including presence of HR-HPV types, positive endocervical margins, HPV persistence and diagnosis of CIN3 [39, 40]. Therefore, women with abnormal screening, regardless of whether they need treatment, should be regularly followed up and examined.

This study has several limitations. First, this online survey was conducted based on a convenience sampling method. Although our large-size sample was collected from seven Chinese geographic regions, respondents mainly resided in cities, limiting the applicability of our findings. Second, the target of 70% set by WHO focuses on the coverage of twice-lifetime screening using a high-performance test. However, our study only investigated whether women had received cervical cancer screening, without considering the quality of screening methods. Thus, this study may overestimate the utilization of cervical cancer screening. Finally, there is a chance of recall bias because the data were self-reported by respondents.

## Conclusion

In conclusion, this study showed the discrepancies between Chinese women’s willingness and screening participation. We found that there is a high willingness among Chinese women aged between 35 to 64 to engage in cervical cancer screenings, but with a low screening rate. The awareness about cervical cancer screening has risen significantly. However, women still have limited knowledge about cervical cancer. The unscientific notion that they have no risk of cervical cancer is the top obstacle for the participation in cervical cancer screening among Chinese women. In order to meet the 70% target set by WHO, comprehensive health education should be enhanced through social media platforms and medical workers. Meanwhile, it is important to promote national free cervical cancer screening with high-performance screening methods.

## Data Availability

The datasets generated and/or analyzed during the current study are not publicly available due to personal information protection, patient privacy regulation, and medical institutional data regulatory policies, etc., but are available from the corresponding author on reasonable request and with permission of the Chinese Academy of Medical Sciences and Peking Union Medical College data sharing committee.

## References

[1] Wang J, Elfström KM, Andrae B, Nordqvist Kleppe S, Ploner A, Lei J (2020). Cervical cancer case-control audit: results from routine evaluation of a nationwide cervical screening program. Int J Cancer.

[2] Andrae B, Kemetli L, Sparén P, Silfverdal L, Strander B, Ryd W (2008). Screening-preventable cervical cancer risks: evidence from a nationwide audit in Sweden. J Natl Cancer Inst.

[3] Schiffman M, Castle PE, Jeronimo J, Rodriguez AC, Wacholder S (2007). Human papillomavirus and cervical cancer. Lancet.

[4] Lynge E, Rygaard C, Baillet MV, Dugué PA, Sander BB, Bonde J (2014). Cervical cancer screening at crossroads. APMIS.

[5] Lynge E (1989). Screening for cancer of the cervix uteri. World J Surg.

[6] World Health Organization. Global Strategy to Accelerate the Elimination of Cervical Cancer. Available from: https://www.who.int/publications/i/item/9789240014107.

[7] Singh D, Vignat J, Lorenzoni V, Eslahi M, Ginsburg O, Lauby-Secretan B (2023). Global estimates of incidence and mortality of cervical cancer in 2020: a baseline analysis of the WHO Global Cervical Cancer Elimination Initiative. Lancet Glob Health.

[8] World Health Organization. Cervical cancer. 2020. Available from: https://www.who.int/news-room/fact-sheets/detail/cervical-cancer.

[9] Arbyn M, Weiderpass E, Bruni L, de Sanjosé S, Saraiya M, Ferlay J (2020). Estimates of incidence and mortality of cervical cancer in 2018: a worldwide analysis. Lancet Glob Health.

[10] Duan R, Qiao Y, Clifford G, Zhao F (2020). Cancer burden attributable to human papillomavirus infection by sex, cancer site, age, and geographical area in China. Cancer Med.

[11] Hahm MI, Chen HF, Miller T, O’Neill L, Lee HY (2017). Why do some people choose opportunistic rather than organized cancer screening? The Korean National Health and Nutrition Examination Survey (KNHANES) 2010–2012. Cancer Res Treat.

[12] Pelullo CP, Cantore F, Lisciotto A, Di Giuseppe G, Pavia M (2021). Organized breast and cervical cancer screening: attendance and determinants in Southern Italy. Cancers (Basel).

[13] Heinävaara S, Sarkeala T, Anttila A (2016). Impact of organised mammography screening on breast cancer mortality in a case-control and cohort study. Br J Cancer.

[14] Di J, Rutherford S, Chu C (2015). Review of the cervical cancer burden and population-based cervical cancer screening in China. Asian Pac J Cancer Prev.

[15] Zhu J, Ge Z, Xia J, Liu Q, Ran Q, Yang Y (2022). Status quo and problem analysis of cervical cancer screening program in China: based on RE-AIM framework. Front Public Health.

[16] Zhang M, Bao H, Wang L, Zhao Z, Huang Z, Zhang X (2021). Analysis of cervical cancer screening and related factors in China. Natl Med J China.

[17] Simayi D, Yang L, Li F, Wang YH, Amanguli A, Zhang W (2013). Implementing a cervical cancer awareness program in low- income settings in Western China: a community-based locally affordable intervention for risk reduction. Asian Pac J Cancer Prev.

[18] Lin W, Huang W, Mei C, Zhong C, Zhu L, Liu P (2022). Pre-procedural anxiety and associated factors among women seeking for cervical cancer screening services in Shenzhen, China: does past screening experience matter?. Front Oncol.

[19] Liu T, Li S, Ratcliffe J, Chen G (2017). Assessing knowledge and attitudes towards cervical cancer screening among rural women in Eastern China. Int J Environ Res Public Health.

[20] Lin W, Chen B, Hu H, Yuan S, Wu B, Zhong C (2021). Joint effects of HPV-related knowledge and socio-demographic factors on HPV testing behaviour among females in Shenzhen. Eur J Public Health.

[21] Setiawan D, Miranti I, Partiwi TD, Puspitasari DA, Ramadhan FN (2022). The willingness for cervical cancer screening among sexually active women in Indonesia: Lesson learned from two districts. Int J Gynaecol Obstet.

[22] Srinath A, van Merode F, Rao SV, Pavlova M. Barriers to cervical cancer and breast cancer screening uptake in low-and-middle-income countries: a systematic review. Health Policy Plan. 2022. 10.1093/heapol/czac104 PMC10089064 36525529

[23] Zhao X, Wang Y, Liu Z, Duan X, Hu S, Wang Y, et al. Knowledge and its influencing factors of cervical cancer screening and human papillomavirus vaccines among 19201 Chinese population. Chin J Cancer Prev Treat. 2022;29(9).

[24] Xu C, Zhang W, Wu M, Zhang S (2011). Knowledge of cervical cancer among 25–54-year-old women in Beijing. J Cancer Educ.

[25] Chua GT, Ho FK, Tung KT, Wong RS, Cheong KN, Yip PS (2020). Sexual behaviors and intention for cervical screening among HPV-vaccinated young Chinese females. Vaccine.

[26] Chan DNS, So WKW (2022). Influential barriers perceived by South Asians in Hong Kong to undergoing cervical cancer screening. Eur J Cancer Care (Engl).

[27] Yang H, Li SP, Chen Q, Morgan C (2019). Barriers to cervical cancer screening among rural women in eastern China: a qualitative study. BMJ Open.

[28] Woo JST, Brotto LA, Gorzalka BB (2009). The role of sexuality in cervical cancer screening among Chinese women. Health Psychol.

[29] Li R, Lewkowitz AK, Zhao FH, Zhou Q, Hu SY, Qiu H (2012). Analysis of the effectiveness of visual inspection with acetic acid/Lugol’s iodine in one-time and annual follow-up screening in rural China. Arch Gynecol Obstet.

[30] Zhao FH, Lin MJ, Chen F, Hu SY, Zhang R, Belinson JL (2010). Performance of high-risk human papillomavirus DNA testing as a primary screen for cervical cancer: a pooled analysis of individual patient data from 17 population-based studies from China. Lancet Oncol.

[31] Zhang J, Zhao Y, Dai Y, Dang L, Ma L, Yang C (2021). Effectiveness of high-risk human papillomavirus testing for cervical cancer screening in China: a multicenter, open-label, randomized clinical trial. JAMA Oncol.

[32] Zhao Y, Bao H, Ma L, Song B, Di J, Wang L (2021). Real-world effectiveness of primary screening with high-risk human papillomavirus testing in the cervical cancer screening programme in China: a nationwide, population-based study. BMC Med.

[33] Hu S, Xu X, Zhang Y, Liu Y, Yang C, Wang Y (2021). A nationwide post-marketing survey of knowledge, attitude and practice toward human papillomavirus vaccine in general population: Implications for vaccine roll-out in mainland China. Vaccine.

[34] Thorburn S, Keon KL, Kue J (2013). Sources of breast and cervical cancer information for Hmong women and men. Women Health.

[35] Tran JH, Mouttapa M, Ichinose TY, Pang JK, Ueda D, Tanjasiri SP (2010). Sources of information that promote breast and cervical cancer knowledge and screening among native Hawaiians in Southern California. J Cancer Educ.

[36] Bogani G, Donato VD, Scambia G, Landoni F, Ghezzi F, Muzii L (2022). Practice patterns and 90-day treatment-related morbidity in early-stage cervical cancer. Gynecol Oncol.

[37] Bogani G, Di Donato V, Scambia G, Raspagliesi F, Chiantera V, Sozzi G (2022). Radical hysterectomy for early stage cervical cancer. Int J Environ Res Public Health.

[38] Cianci S, Tarascio M, Arcieri M, La Verde M, Martinelli C, Capozzi VA (2023). Post treatment sexual function and quality of life of patients affected by cervical cancer: a systematic review. Medicina (Kaunas).

[39] Giannini A, Di Donato V, Sopracordevole F, Ciavattini A, Ghelardi A, Vizza E (2023). Outcomes of high-grade cervical dysplasia with positive margins and HPV persistence after cervical conization. Vaccines (Basel).

[40] Bogani G, Lalli L, Sopracordevole F, Ciavattini A, Ghelardi A, Simoncini T (2022). Development of a nomogram predicting the risk of persistence/recurrence of cervical dysplasia. Vaccines (Basel).

